# Validation of the factor structure of the Experiences Questionnaire using Exploratory Graph Analysis

**DOI:** 10.3389/fpsyg.2023.1250802

**Published:** 2023-11-15

**Authors:** Lena Rader, Barbara Drueke, Saskia Doreen Forster, Siegfried Gauggel, Verena Mainz

**Affiliations:** Institute of Medical Psychology and Medical Sociology, University Hospital RWTH Aachen, Aachen, Germany

**Keywords:** decentering, metacognition, experiences questionnaire, exploratory graph analysis, psychometric properties

## Abstract

**Introduction:**

Decentering describes the ability to shift the focus away from one’s subjective experience onto the experience itself. The Experiences Questionnaire (EQ) is a self-report measure that was developed to systematically assess changes in Decentering ability. Although several studies show the validity of the questionnaire, there are discrepancies between the factorial structure of the Decentering scale of the EQ (EQ-D) found in the initial study (one factor) and other studies (two factors). Therefore, the current study aimed to assess the dimensionality of the EQ-D using Exploratory Graph Analysis (EGA).

**Methods:**

In total, 1,100 participants were recruited online (790 female, 307 male, 3 non-binary; age 18 to 65 years). Participants completed the EQ and the Rosenberg Self-esteem scale (RSES).

**Results:**

The bootstrapped EGA results revealed a two-dimensional structure of the EQ-D (Factor 1: Distanced Perspective, DP; Factor 2: Accepting Self-perception, AS) with high structural and item stability (all items > 0.70). The two dimensions of the EQ-D showed a high internal consistency (DP: *ω* = 0.74; AS: *ω* = 0.86) and discriminant validity with the rumination items of the EQ. Furthermore, a high convergent validity of the EQ was established, as the AS factor exhibited a significantly stronger correlation with self-esteem than the DP factor (*z* = 7.98, *p* < 0.001), which aligns with theoretical considerations suggesting that the AS factor encompasses aspects of self-compassion alongside decentering. We also found measurement invariance of the DP and AS factor across age, gender and country but not for education.

**Discussion:**

These results support the EQ’s validity, demonstrated in a larger sample with a new methodology, aligning with existing two-factor decentering models literature.

## 1. Introduction

Decentering is a subject of growing interest in both research and clinical practice. It describes the ability to take a step back from a situation and to shift the focus away from one’s subjective experience onto the experience itself (Safran and Segal, 1996). Through this process, people can take a more objective, non-judgmental stance toward themselves (Safran and Segal, 1996; Fresco et al., 2007). Decentering is also a core mechanism in psychological interventions for anxiety and depression (Bennett et al., 2021) and a key component in Mindfulness-Based Cognitive Therapy (MBCT; Segal et al., 2018). It has been associated with enhanced treatment outcomes, including for instance a decreased risk of relapse in individuals with depression, as well as improved emotion regulation resulting in reduced anxiety and worry in patients with generalized anxiety disorder (Hayes-Skelton and Lee, 2018; O’Toole et al., 2019; Moore et al., 2022). King and Fresco (2019) suggest that the positive effects of decentering may be mediated by changes in both within-network and between-network connectivity associated with attention and executive functions, including the salience network, frontotemporal control network, and default mode network. In summary, it can be concluded that decentering has been shown in numerous studies to be an important metacognitive ability for reducing psychological distress in patients with mental disorders and enhancing overall well-being in the general population (Josefsson et al., 2014).

Fresco et al. (2007) developed the Experiences Questionnaire (EQ) which systematically assesses changes in decentering ability before versus after intervention. In an initial validation study, the authors found an acceptable fit for a one-factor solution in student samples and a clinical sample (Fresco et al., 2007). The EQ is now one of the most frequently used questionnaires for the assessment of decentering in cross-sectional studies (Naragon-Gainey et al., 2020). It has also been adapted in Spanish (Soler et al., 2014), Portuguese (Gregório et al., 2015), Hebrew (Hadash et al., 2017) and German (Gecht et al., 2014). While Soler et al. (2014) and Gregório et al. (2015) both found a good fit for the one-factor model, Gecht et al. (2014) proposed a two-factor model for the EQ-D with the factors *Distanced Perspective* (DP) and *Accepting Self-perception* (AS). Similarly and more recently, Hadash et al. (2017) examined the factorial structure of decentering across different self-report measures. They conducted an exploratory and confirmatory factor analysis (CFA) with five self-report measures of decentering. The authors identified the optimal configuration as a two-factor solution, labeling these factors as *Intentional Decentered Perspective* and *Automatic Reactivity to Thought Content*. The authors noted that the items from the EQ-D were not retained in the final factor solution. They argue that the EQ-D also measures processes that are conceptually distinct from decentering such as reduced reactivity to feelings and self-compassion, next to metacognitive processes of decentering such as meta-awareness (Hadash et al., 2017). Naragon-Gainey and DeMarree (2017) found a similar two-factorial structure across five self-report measures of decentering using exploratory structural equation modelling. An item-level analysis, again, revealed a two-factorial structure with the factors *Observer Perspective* and *Reduced Struggle with Inner Experience*. Most items of the EQ-D significantly loaded on the *Observer Perspective* factor.

Regarding to the above findings, the aims of the current study were to, first, validate the one-factorial model originally proposed by Fresco et al. (2007) using CFA to assess its model fit in a large, more representative sample. However, and considering the heterogeneous results with regard to the dimensionality of decentering found in previous studies, the second aim was to further explore the factor structure of the EQ-D using a novel technique called exploratory graph analysis (EGA). EGA is currently proposed as the most accurate method in estimating the number of dimensions in a data set (Golino and Epskamp, 2017). A simulation study by Golino et al. (2020) recently demonstrated the superiority of EGA (87.91% accuracy) compared to parallel analysis (83.01% accuracy) in estimating the number of factors in a simulated data set. EGA assesses the dimensionality based on a regularized partial correlation matrix as opposed to a variance–covariance matrix in conventional factor analyses. First, a graphical least absolute shrinkage and selection operator (GLASSO) is applied to a correlation matrix, which penalizes variables that are weakly correlated. This results in a sparse network showing the items (nodes) and their (regularized partial) correlations (edges). In such a network, item proximity holds significance, meaning that highly correlated items are positioned in closer proximity to each other, in contrast to weakly associated items. Also, the extend of association between two items can be expressed by more pronounced edges (i.e., the thicker the lines, the higher the correlation between two nodes). The advantage of applying the GLASSO is that clusters of highly correlated items can be more easily identified and the sparse correlation matrix can be more easily interpreted. The EGA also allows a graphical representation of the factor structure or dimensions in a network plot, which additionally simplifies and objectifies the interpretation. The number of dimensions in the data can then be estimated using a walktrap algorithm. The walktrap algorithm in combination with the GLASSO has been shown to be one of the most accurate and least biased community detection algorithms (Christensen et al., 2020a). Another advantage of the method is the possibility of checking the stability of the results by bootstrapping (Christensen and Golino, 2021). This allows the robustness (reliability) of the results to be calculated at scale and item level (a more detailed description is provided in the Methods section).

Next, to estimating the number of dimensions in the EQ, the current study aimed to assess and validate findings concerning the measurement invariance of the EQ-D using EGA. A study by Naragon-Gainey et al. (2020) recently demonstrated measurement invariance of the EQ-D across age, gender, race/ethnicity, and meditation experience in three samples (students, community members, and clinical participants) using structural equation modeling. In addition, the current study examined the validity of the EQ-D by assessing its associations with the related concepts of rumination and self-esteem. Rumination is defined as negative perseverant thought pattern about the past, present and future and is a transdiagnostic mechanism associated with different mental disorders such as depression and anxiety (Watkins and Roberts, 2020). Decentering is proposed to foster self-reflection through a decentered self-focused attention, thereby reducing rumination and depressive symptoms (e.g., Mori and Tanno, 2015; Wolkin, 2015). In previous studies, correlations between decentering and rumination for instance ranged from −0.32 to −0.70 with a significance of *p* < 0.05 (Fresco et al., 2007; Naragon-Gainey and DeMarree, 2017). Therefore, we expected to find a negative association between rumination and decentering replicating the results of previous studies. With regard to self-esteem, studies on depression and anxiety suggest that the positive effects of mindfulness, a concept closely related to decentering, are mediated through self-esteem (Bernstein et al., 2015; Bajaj et al., 2016). Self-esteem is defined as the value a person ascribes to him-or herself (Baumeister and Finkel, 2010). People with high self-esteem are suggested to have better access to personal resources (Harris et al., 2019), supposedly through a decentered self-focused attention. Moreover, decentering has been shown to buffer the effect of negative feedback on peoples’ self-esteem, indicating that the higher a person’s decentering abilities the better a person can cope with negative feedback and restore their self-esteem (Lyddy et al., 2022). Naragon-Gainey and DeMarree (2017) for instance reported correlations between decentering and self-esteem ranging from 0.47 to 0.67 with a significance of *p* < 0.01. Based on these findings, a positive association between decentering and self-esteem was expected.

In summary, the present study aims to validate the factorial structure of the EQ-D through CFA and EGA. Based on previous research findings, we anticipate a one-factor structure with a general decentering factor as proposed by Fresco et al. (2007) or a two-factor structure with factors resembling those found in other studies (i.e., *Accepting Self-perception* & *Distanced Perspective;* Gecht et al., 2014). Additionally, the measurement invariance of the EQ-D will be examined using EGA. Consistent with the results of Naragon-Gainey et al. (2020), we expect measurement invariance across all tested variables (age, gender, education, country). Furthermore, the validity of the EQ-D will be assessed through correlations with relevant constructs such as rumination and self-esteem. Based on previous studies, we anticipate a negative correlation between the EQ-D and the rumination items of the EQ, and a positive correlation between the EQ-D and self-esteem.

## 2. Methods

### 2.1. Participants

The sample comprised 1,100 participants, of which 790 identified as female, 307 as male, and 3 as non-binary. The participants age ranged from 18 to 65 years (*M* = 47, *SD* = 12). The school degrees were distributed as follows: 51% high school degree, 41% university degree, 2% middle school degree, and 6% another degree. Exclusion criteria were an age younger than 18 or older than 65 and not being fluent in English. The study was conducted as part of a larger research project which was pre-registered (doi:10.23668/psycharchives.5105).

### 2.2. Material

#### 2.2.1. Decentering questionnaire

Decentering was assessed using the Experiences Questionnaire (EQ) which is a self-report measure developed by Fresco et al. (2007) to assess subjective changes in decentering. The EQ contains 14 Decentering items (EQ-D) and 6 Rumination items (item 1, 4, 7, 11, 13, and 19). In their initial validation study, Fresco et al. (2007) found a one-factorial structure with 11 decentering items (item 3, 6, 8, 10, 12, 14, 15, 16, 17, 18 and 20). The items are answered on a scale from 1 (“Never”), to 5 (“All the time”). A high score on the Decentering items suggests a high Decentering tendency. An example item from the EQ-D is *I can separate myself from my thoughts and feelings* (item 10). The EQ-D showed a high internal consistency in the current sample (*ω* = 0.88) which aligns with the findings by Fresco et al. (2007; *α* = 0.83–0.90). The rumination subscale of the EQ also showed a high internal consistency (*ω* = 0.82).

#### 2.2.2. Self-esteem questionnaire

Self-esteem was assessed using the 10-item Rosenberg Self-esteem scale (RSES; Rosenberg, 1965). The items are answered on a scale from 1 (“strongly agree”) to 4 (“strongly disagree”). Item 1, 3, 4, 7, and 10 were reverse-coded so that a high score indicates high self-esteem. An example item from the RSES is *I feel that I have a number of good qualities* (item 3). The RSES showed also showed a high internal consistency in the current sample (*ω* = 0.94).

### 2.3. Procedure

#### 2.3.1. Data collection

Participants were recruited via the online platform Qualtrics (Qualtrics Labs Inc., Provo, Utah).^1^ Prior to answering the questionnaires, participants were asked for sociodemographic data regarding their age, gender, native language, nationality, and level of education (school degree). Participants then answered the EQ and RSES in randomized order.

#### 2.3.2. Data integrity checks

Two data integrity checks were conducted – an attention check midway through the questionnaire and a post-hoc check for straight-liners. The attention check was built in the online survey and asked participants to answer the following item midway through the questionnaire: *Please choose the answer option “all the time” for this item*. If participants did not indicate the answer option “all the time” the survey was terminated at this point and participants were automatically screened out. The straight-liner check was conducted once the data collection was completed. Participants were defined as straight-liners if they gave the same answer option on all 20 items and thus had a variance of zero. No participant had to be excluded based on the post-hoc data integrity check. The final sample only included participants who passed both data integrity checks.

### 2.4. Data analysis

We used R Studio (R Core Team, 2022) for all analyses. The raw data supporting the conclusions of this article and code for the data analysis associated with the current submission are available from the repository of the Open Science Framework.^2^

#### 2.4.1. Confirmatory factor analysis

The one-factorial model of the EQ-D proposed by Fresco et al. (2007) was tested in a Confirmatory Factor Analysis (CFA) using maximum likelihood estimation. We included the same correlated residuals as in Fresco et al. (2007). Moreover, a post-hoc analysis which tested the model fit of the original one-factor model in a subsample of university students (*n* = 447) was conducted. The aim of this analysis was to find out whether the previous findings on the 1-factorial structure of the EQ-D could possibly be explained by the selectivity of the student sample.

Model fit was examined using measures of absolute and relative fit. We used the $\chi^{2}$-test (H0: the empirical variance–covariance matrix is equal to the hypothesized, model-implied variance–covariance matrix), Root Mean Square Error of Approximation (RMSEA), Standardized Root Mean Square (SRMR) and Comparative Fit Index (CFI) to assess absolute fit. In addition, the $\chi^{2}$/degrees of freedom (df) ratio was calculated since the $\chi^{2}$-test has been shown to be sensitive to sample size (Kline, 2023). A $\chi^{2}$/df ratio < 2, and RMSEA and SRMR values <0.05 indicate good fit (Cangur and Ercan, 2015). The CFI tests whether the hypothesized model fits the data better compared to an independence model. CFI > 0.95 indicates good fit (Jackson et al., 2009). Based on a simulation study, Golino et al. (2020) advised to interpret CFA model fit indices as relative rather than absolute indices. We also used the Akaike Information Criterion (AIC) and the Total Entropy Fit Index (TEFI) to assess the relative fit of different models of the EQ. The AIC indicates the goodness of fit based on the maximum likelihood value while taking into account the number of parameters estimated in the model. Lower AIC values indicate better model fit. The TEFI indicates the degree of uncertainty whereby lower values indicate lower uncertainty meaning that a given factor structure represents the organization of these variables well (Golino et al., 2021). We used the R package lavaan (Rosseel, 2012) to conduct the CFA and the EGAnet package (Hudson and Alexander, 2022) to calculate the TEFI.

#### 2.4.2. Exploratory graph analysis

The dimensionality of the EQ-D was further assessed by means of an Exploratory Graph Analysis (EGA; Golino et al., 2020) using the EGAnet package (Hudson and Alexander, 2022) in R. We included all 14 decentering items of the EQ in the EGA.

First, we conducted a redundancy analysis to identify local dependencies (e.g., highly correlated items because of wording effects). Whereas traditional factor models assume that questionnaire items measure a common latent variable, network models view items as causally autonomous (Christensen et al., 2020a,b). Redundancies (such as local dependencies) can negatively affect the estimation of dimensions and should therefore be considered prior to conducting an EGA.

Next, a network was estimated using the Graphical Least Absolute Shrinkage and Selection Operator (GLASSO). Applying the GLASSO results in a regularized partial correlation matrix that is shown as a Gaussian Graphical Model where nodes represent items and edges represent the partial correlations between two items. The EGA then applies a walktrap algorithm to detect the number of communities (i.e., highly correlated items; Golino and Epskamp, 2017). Since the results of the EGA can be biased due to sampling variability, Christensen and Golino (2021) advise to assess the robustness of the EGA results using bootstrapping. A non-parametric bootstrapping (resampling) with 1,000 iterations was applied in the current study.

#### 2.4.3. Reliability

In EGA, the reliability of a network is estimated based on structural and item stability (Christensen and Golino, 2021). The structural consistency is an indicator of the stability of the extracted dimensions across all bootstrap samples (i.e., it gives the percentage of number of dimensions found in each bootstrap sample). Item stability on the other hand indicates “the robustness of each item’s placement within each empirically derived dimension” (Christensen and Golino, 2021, p. 4), i.e., it is assessed whether the items are allocated to the same dimension across all bootstrap iterations. According to Christensen and Golino (2021), items displaying low item stability (< 0.70) should be removed. Therefore, items with low stability were removed iteratively and the analysis was repeated each time without the removed items. In addition, the reliability of the dimensions found in the EGA was assessed using McDonald’s omega (ω).

#### 2.4.4. Associations between decentering, rumination and self-esteem

To assess the discriminant validity of the decentering subscale of the EQ (EQ-D), an EGA with the decentering and rumination items of the EQ was conducted. It was expected that the rumination items would form an independent dimension in the EGA, thereby demonstrating discriminant validity to the decentering items. The validity of the EQ-D was further assessed by examining the associations between the decentering factor(s) found in the EGA, rumination and self-esteem using Pearson’s r. Based on the results of previous studies, rumination (assessed with the 6 rumination items of the EQ) and self-esteem (assessed with the RSES) were expected to be negatively, resp. positively associated with decentering. A Fisher’s z-test was used to test whether the correlations between the decentering dimensions found in the EGA and rumination, respectively self-esteem, were significantly different.

#### 2.4.5. Measurement invariance across age, gender, education and country

Measurement variance was assessed across age (young (</ = 49 years) vs. old (>49 years)), gender (male vs. female), education (high school vs. university degree) and country (United Kingdom vs. United States). The number of participants per group can be found in Table 1. Configural invariance can be assumed when the EGA recovers the same dimensional structure across all groups (i.e., the same nodes have been partitioned into the same communities for all groups; Jamison et al., 2022). The configural invariance of the EQ was assessed by comparing the bootstrapped EGA results across the sociodemographic groups described above.

**Table 1:** Metric invariance of the EQ-D across gender, age, country and education using standardized network loadings based on bootstrapped EGA results.

EQ item	Gender					Age					Country					Education				
	Females (*n* = 790)		Males (*n* = 307)		*p*	Younger (*n* = 567)		Older (*n* = 532)		*p*	UK (*n* = 550)		USA (*n* = 550)		*p*	High school (*n* = 562)		University (*n* = 447)		*p*
	DP	AS	DP	AS		DP	AS	DP	AS		DP	AS	DP	AS		DP	AS	DP	AS	
3	0.19	0.29	0.13	0.24	0.280	0.15	0.24	0.20	0.31	0.296	0.11	0.31	0.17	0.29	0.594	0.14	0.31	0.18	0.22	–
5	0.04	0.17	0.10	0.23	0.422	0.09	0.19	0.03	0.18	0.694	0.09	0.22	0.07	0.19	0.294	0.07	0.17	0.10	0.15	–
6	0.01	0.28	0.06	0.30	0.592	0.02	0.29	0.08	0.27	0.444	0.06	0.26	0.03	0.29	0.618	0.01	0.28	0.05	0.26	–
8	0.04	0.14	0.04	0.18	0.204	0.10	0.18	0.02	0.11	0.096	0.04	0.15	0.04	0.15	0.820	0.04	0.18	0.03	0.14	–
9	0.06	0.42	0.10	0.33	0.294	0.05	0.36	0.07	0.45	**0.026**	0.06	0.41	0.05	0.41	0.788	0.07	0.41	0.04	0.43	–
10	0.08	0.35	0.03	0.33	0.520	0.05	0.36	0.07	0.31	0.280	0.08	0.31	0.05	0.34	0.402	0.06	0.30	0.09	0.35	–
15	0.17	0.21	0.13	0.13	**0.046**	0.18	0.22	0.12	0.19	0.948	0.12	0.16	0.15	0.22	0.194	0.13	0.20	0.18	0.23	–
16	0.33	0.05	0.27	0.19	0.308	0.34	0.11	0.27	0.07	0.264	0.30	0.11	0.32	0.08	0.540	0.32	0.08	0.30	0.08	–
17	0.18	0.15	0.24	0.06	0.256	0.20	0.12	0.20	0.13	0.760	0.16	0.15	0.21	0.13	0.236	0.22	0.14	0.13	0.13	–
18	0.38	0.00	0.38	0.08	0.742	0.32	0.07	0.42	0.02	**0.040**	0.41	0.01	0.39	0.01	0.668	0.42	0.00	0.35	0.05	–
20	0.24	0.10	0.30	0.07	0.326	0.29	0.09	0.20	0.09	0.056	0.26	0.09	0.25	0.09	1.000	0.25	0.09	0.25	0.12	–

Note: Younger, participants who are 49 years or younger; older, participants who are older than 49 years; DP, Distanced Perspective; AS, Accepting Self-perception; since the EQ-D did not show configural invariance for education, metric invariance was not assessed and p-values cannot be reported; values of *p* < 0.05 are marked as bold.

To assess metric invariance, the network loadings of the two groups (e.g., young vs. old) are compared using permutation testing. Specifically, as for configural invariance, a network is estimated for each group using bootstrapped EGA results. Then, a test statistic is calculated for each item by assessing the difference between the network loadings of each group. This test statistic is then compared to a null distribution in which there is no association between the network loadings and group membership (e.g., age). In the final step, the test statistic is compared to its null distribution for each item at *α* = 0.05 (Jamison et al., 2022).

## 3. Results

### 3.1. Confirmatory factor analysis

The CFA revealed that the 1-factor model fits the data poorly ($\chi^{2}$(44) = 324.56, *p* < 0.001, $\chi^{2}$/df = 7.37, RMSEA = 0.076, SRMR = 0.058, CFI = 0.764). When the residual correlations proposed by Fresco et al. (2007) were added, the model fit improved ($\chi^{2}$(41) = 183.94, *p* < 0.001, $\chi^{2}$/df = 4.48, RMSEA = 0.56, SRMR = 0.045, CFI = 0.880). However, the values for $\chi^{2}$/df, RMSEA and CFI still fell below the recommended cut-off ($\chi^{2}$/df < 2; RMSEA < 0.05; CFI > 0.95). The post-hoc analysis including only the university students revealed an acceptable fit for the 1-factor model ($\chi^{2}$(41) = 81.13, *p* < 0.001, $\chi^{2}$/df = 1.97, RMSEA = 0.047, SRMR = 0.043, CFI = 0.915). The results of these analyses suggest that the 1-factorial structure cannot be generalized to the population level. Table 2 shows the model fit indices of the 1-factor model proposed by Fresco et al. (2007) and the factor models of the EQ-D proposed by the EGA.

**Table 2:** Model fit indices of different factor models of the EQ-D.

Model	χ^2^	*df*	*p*	χ^2^/*df*	CFI	RMSEA	SRMR	AIC	TEFI
Fresco^1^	324.56	44	<0.001	7.37	0.764	0.076	0.058	29884.05	0
Fresco^2^	183.94	41	<0.001	4.48	0.880	0.056	0.045	29653.31	-
Fresco^2^_uni_	81.13	41	<0.001	1.97	0.915	0.047	0.043	11448.23	-
EGA^1^	214.97	64	<0.001	3.35	0.880	0.046	0.042	35805.98	−7.11
EGA^2^	152.81	53	<0.001	2.88	0.917	0.041	0.037	32824.11	−6.67
EGA^3^	131.75	43	<0.001	3.06	0.924	0.043	0.037	30141.59	−6.09

Note: Fresco^1^, 1-factor model by Fresco et al. (2007) without correlated residuals; Fresco^2^, model by Fresco et al. (2007) with original correlated residuals; Fresco^2^_uni_, model by Fresco et al. (2007) with original correlated residuals only with university students (*n* = 447); EGA^1^, EGA factor structure without item 14 (item 14 excluded because of redundancy); EGA^2^, EGA factor structure without item 14 and 2 (item 2 excluded because of low stability); EGA^3^, EGA factor structure without item 14, 2 and 12 (item 12 excluded because of low discriminant validity).

### 3.2. Exploratory graph analysis

We further investigated the dimensionality of the EQ-D using EGA. The redundancy analysis revealed a local dependency between item 3 (*I am better able to accept myself as I am*) and 14 (*I can treat myself kindly*). Note, that, interestingly, Fresco et al. (2007) included a residual correlation between item 3 and 14, suggesting that these two items share some error variance. In consequence, item 14 was excluded from further analyses since it had a smaller variance compared to item 3 (item variances as well as descriptive statistics and items contents of the EQ items can be found in Supplementary Table S1).

The GLASSO estimation revealed a one-dimensional structure of the EQ-D. However, the bootstrapped EGA results suggested two dimensions in 79.5% of the iterations, followed by three factors (18.1%), four factors (2.2%) and five factors (0.2%), strongly pointing toward a two-dimensional structure of the EQ-D. An inspection of the item stability showed that most of the items showed a high stability across all bootstrap iterations (ranging from 0.75–1), only item 2 showed a low stability (0.68). The analysis was then repeated without item 2. The typical graph of the bootstrapped EGA and the item stability plot (including item 2) can be found in Supplementary Figures S1, S2.

**Figure 1:**
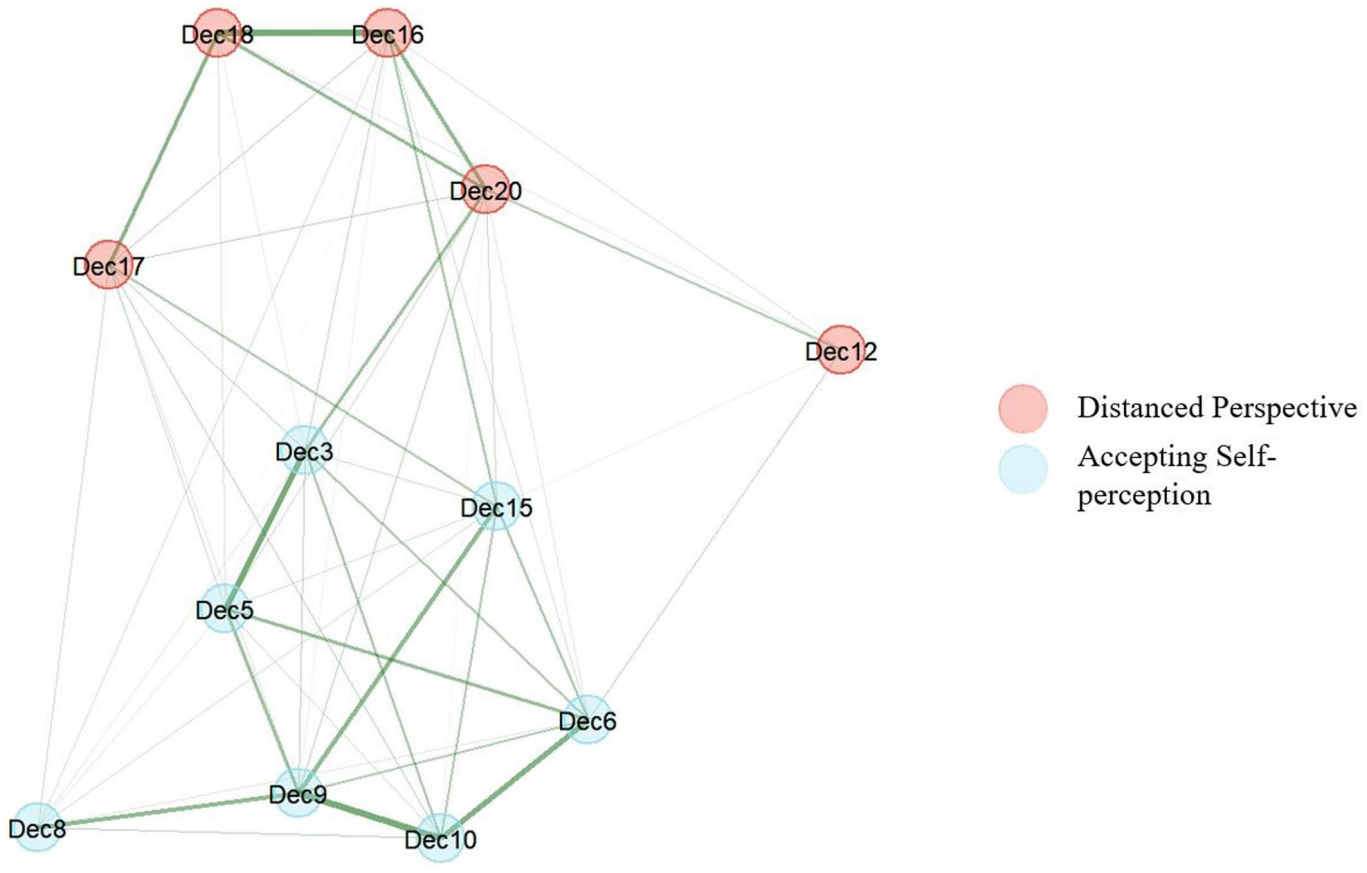
Typical graph of the EQ-D across all 1,000 bootstrap samples of EGA results (excl. Item 14 and 2).

The GLASSO and the bootstrapped EGA results (excluding item 14 and 2) again suggested a two-dimensional structure of the EQ-D (see Figure 1). Based on the item contents, factor 1 and 2 were named after the factors proposed by Gecht et al. (2014). Factor 1 was named *Distanced Perspective* (DP) as it was comprised of items such as *I can actually see that I am not my thoughts* (item 17) or *I view things from a wider perspective* (item 20). Factor 2 was named *Accepting Self-perception* (AS) as it was comprised of items such as *I am better able to accept myself* (item 3) or *I am kinder to myself when things go wrong* (item 5).

### 3.3. Reliability

**Figure 2:**
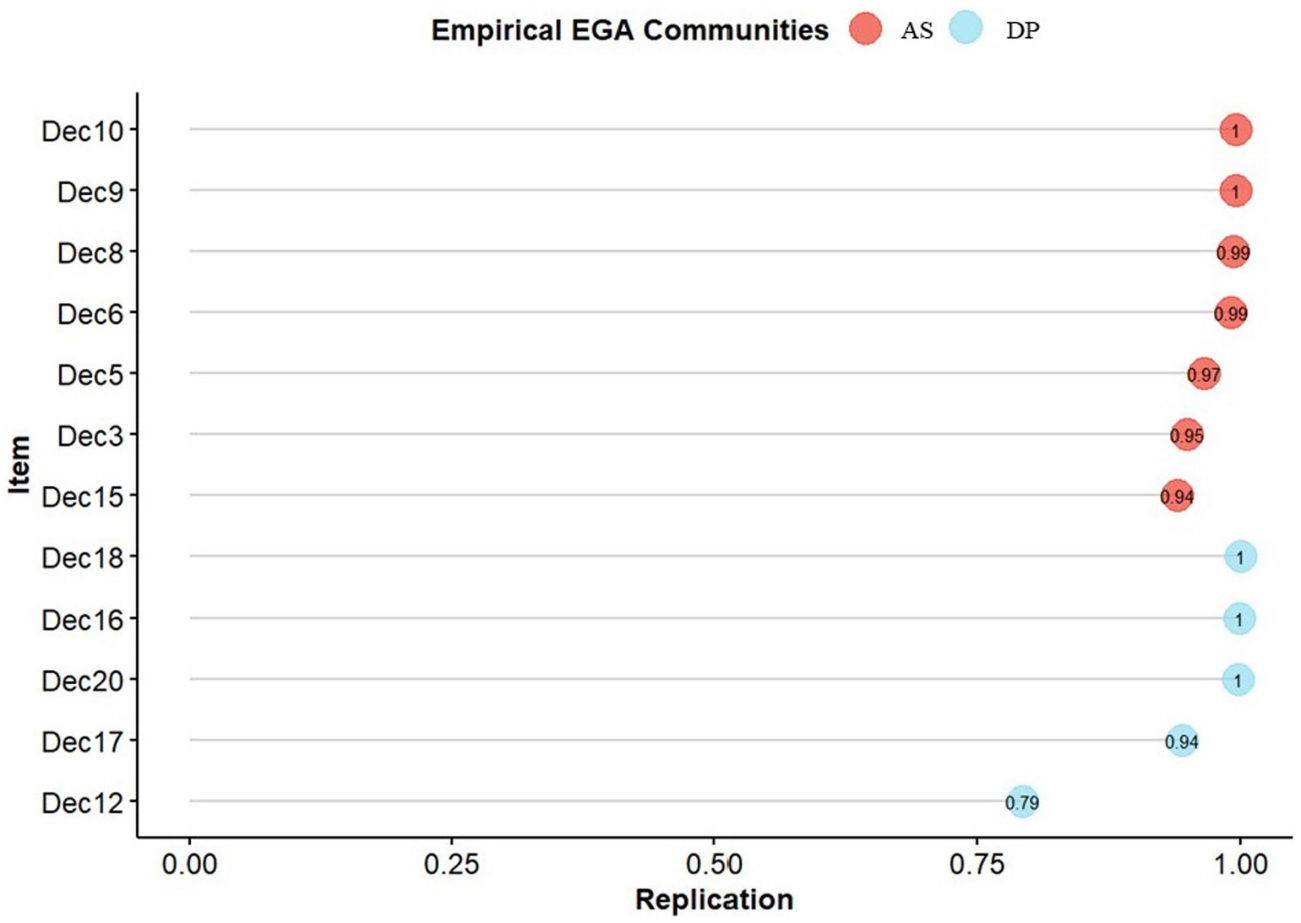
Item stability plot of the EQ-D based on bootstrapped EGA results (excl. Item 14 and 2). AS, Accepting Self-perception; DP, Distanced Perspective.

The two dimensions of the EQ-D shown in Figure 1 showed a high structural consistency as they were replicated in 92.1% of the bootstrap samples. The item stability was also high (> 0.70 for all items; see Figure 2). In addition, McDonald’s omega indicated acceptable to high reliability of the two dimensions (*DP*: *ω* = 0.74; *AS*: *ω* = 0.86).

### 3.4. Associations between decentering, rumination and self-esteem

**Figure 3:**
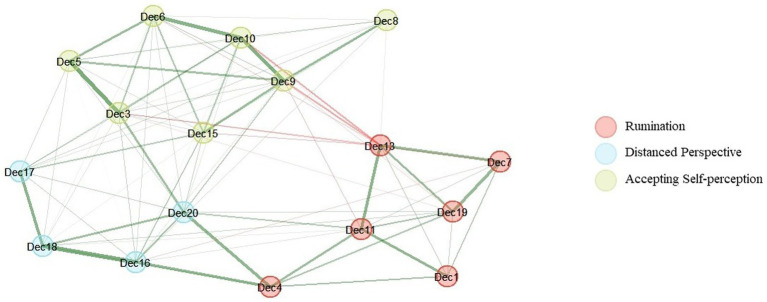
Network plot based on bootstrapped EGA results with decentering and rumination items of the EQ (excl. Item 14, 2, and 12).

The bootstrapped EGA results using the decentering (excl. item 14 and 2) and rumination items revealed a 3-dimensional structure. Item 3, 5, 6, 8, 9, 10 and 15 (*Accepting Self-perception*) and item 16, 17, 18 and 20 (*Distanced Perspective*) formed separate clusters with identical allocation of items as in the previous analysis. However, item 12 which was previously allocated to DP factor was now allocated to the rumination cluster (see Figure 3). Item 12 (*I can take time to respond to difficulties*) thus demonstrated poor discriminant validity. Item 12 also showed the lowest item reliability in the previous analysis (0.79). It was thus decided to exclude item 12 from the following analyses.

Moreover, self-esteem was significantly positively associated with the *Accepting Self-perception* (*r* = 0.65, *p* < 0.001) and the *Distanced Perspective* factor (*r* = 0.48, *p* < 0.001). The *Accepting Self-perception* factor was significantly higher associated with self-esteem than the *Distanced Perspective* (*z* = 7.98, *p* < 0.001). The rumination factor was significantly negatively associated with the *Accepting Self-perception* (*r* = −0.31, *p* < 0.001) but not the *Distanced Perspective* factor (*r* = 0.05, *p* = 0.08).

### 3.5. Measurement invariance across age, gender, education and country

**Figure 4:**
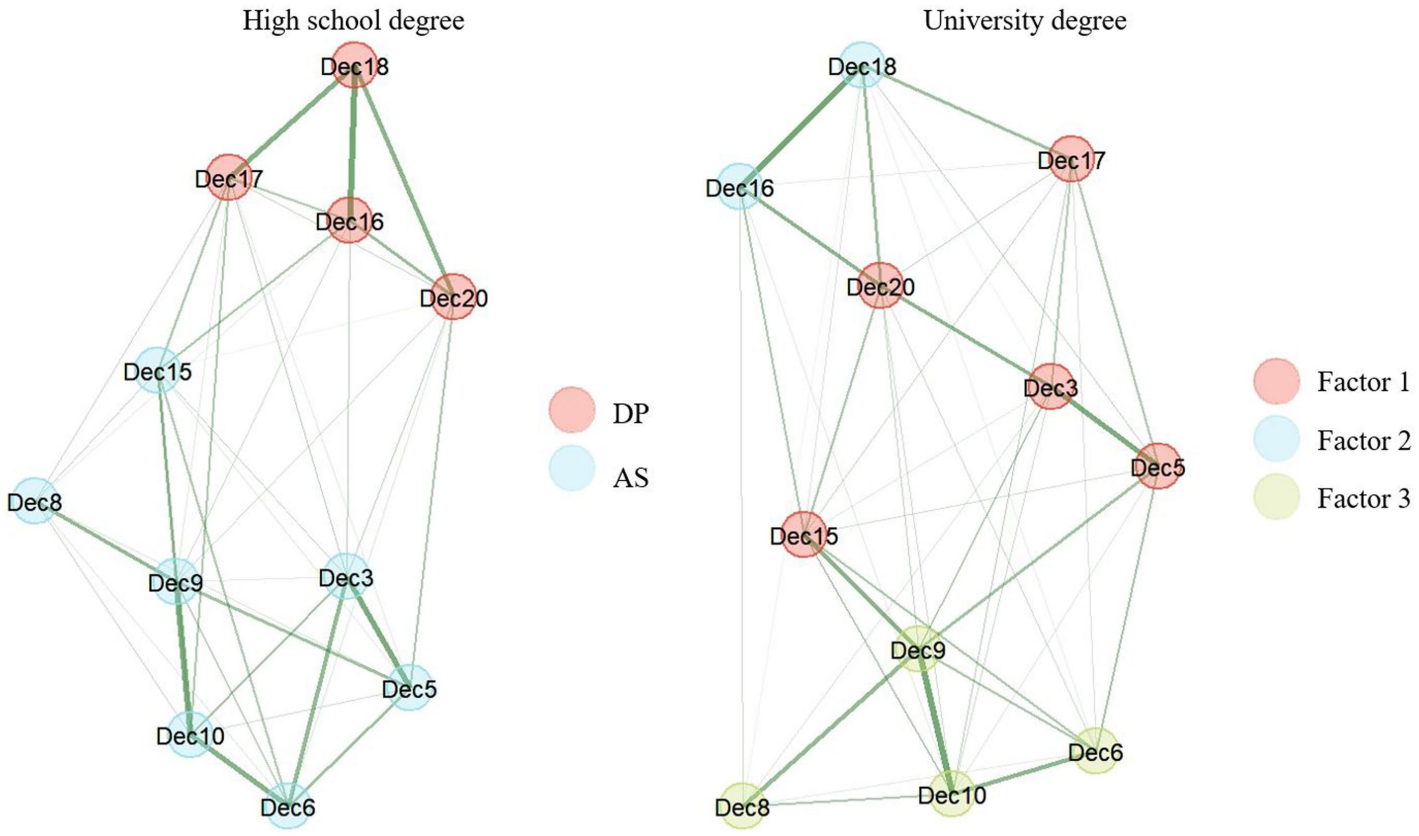
Configural variance of the EQ-D across education based on the bootstrapped EGA results. DP, Distanced Perspective; AS, Accepting Self-perception.

The EQ-D demonstrated configural invariance for age, gender, and country but not for education. For education, different network models were found. Whereas we found the same two-dimensional structure previously shown for participants with high school degree (*n* = 562) we found a three-dimensional structure for participants with university degree (*n* = 447; see Figure 4). In this 3-dimensional structure, factor 1 was comprised of item 3, 5, 15, 17 and 20, factor 2 of item 16 and 18, and factor 3 of item 6, 8, 9, and 10. The AS factor of the two-factor solution of the EQ-D is most likely to be found in factor 3 of the three-factor solution. Factor 3 included the items of the AS factor concerned with reduced reactivity to thoughts and feelings (e.g., item 8: *I am not so easily carried away by my thoughts and feelings*). The DP factor was not found in the 3-factor solution. Two items of the DP factor (items 16 and 18) are found in factor 2, factor 1 contains both AS and DP items. As configural invariance depicts a prerequisite to assess metric invariance, *p*-values for education are not reported in Table 1.

In a next step, the metric invariance of the EQ-D was tested across gender, age and country. The network loadings across gender and age were only partially invariant. For gender, the network loadings of item and 15 (*I can observe unpleasant feelings without being drawn into them*) were found to differ significantly. For age, significant differences in the network loadings were found for item 9 (*I notice that I do not take difficulties so personally*) and 18 (*I am consciously aware of a sense of my body as a whole*; see Table 1). The network loadings were found to be metrically invariant across the two country samples (United Kingdom vs. United States).

## 4. Discussion

The aim of the current study was to validate the factorial structure of the decentering subscale of the Experiences Questionnaire (EQ-D) using CFA as originally applied by Fresco et al. (2007) and, moreover, by means of an EGA. In addition, the associations between decentering, rumination and self-esteem were investigated. The results of the CFA replicating the 1-factor solution by Fresco et al. (2007) revealed poor model fit, suggesting that the 1-factorial structure cannot be generalized to the population level. Interestingly, conducting the CFA focusing on the university student sample, an improved model fit of the original one-factor model proposed by Fresco et al. (2007) emerged.

Applying the EGA on the total sample we found two dimensions for the EQ-D with good validity and reliability. The two-dimensional structure demonstrated measurement invariance for age, gender and country, but not for education. This finding is especially relevant since the EQ-D has frequently been validated with student samples similar to Fresco et al. (2007). The results of the current study suggest that the items might function in a different way for people with high school vs. university degree, which limits the generalizability of the results. For people with university degree we found a three-dimensional structure that did not even closely resemble the two-dimensional structure found for the remaining sample. Taken together, the data of the study at hand suggest that the good fit of the one-factor model found in previous studies (e.g., Fresco et al., 2007; Soler et al., 2014; Gregório et al., 2015) might apply for certain samples (e.g., young, highly educated) rather than resembling the characteristics of decentering in its entirety, which hampers the generalizability of a one-factor model. The current study investigated a large sample with a broader age range. The bootstrapped EGA resulted in a two-dimensional structure which is consistent with other studies suggesting, for one, two factors in the EQ-D (Gecht et al., 2014) and, moreover, two factors across different self-report measures of decentering (Hadash et al., 2017; Naragon-Gainey and DeMarree, 2017). In the following we would like to shortly discuss the parallels and peculiarities of our findings in light of the literature.

In the final solution, the EQ-D showed a two factorial structure with high structural and item stability based on the bootstrapped EGA results. The two dimensions found in the EQ-D were named after the factors proposed by Gecht et al. (2014) based on the item contents, respectively. Factor 1 was named *Distanced Perspective* (DP) and comprised 4 items. Factor 2 was named *Accepting Self-perception* (AS) and comprised 7 items. Hadash et al. (2017) also found a two-factorial structure (*Intentional Decentered Perspective, Automatic Reactivity to Thought Content*) across different self-report measures of decentering. Similarities between the factors can for instance be found with regard to item 17 (*I can actually see that I am not my thoughts*) in the DP factor, respectively item 10 (*I can separate myself from my thoughts and feelings*) in the AS factor. However, the authors proposed that the EQ-D also measures aspects that are conceptually distinct from decentering such as self-compassion (Hadash et al., 2017). In the current study, the AS factor also included items such as *I am better able to accept myself* (item 3) and *I am kinder to myself when things go wrong* (item 5). These items can be said to measure concepts such as reduced reactivity to feelings and self-acceptance, aspects that according to Hadash et al. (2017) are not necessarily subsumed under decentering. In our model, the AS factor also encompasses items like *I can separate myself from my thoughts and feelings* (item 10) and *I can observe unpleasant feelings without being drawn into them* (item 15). However, it is worth noting that these items more closely assess decentering, as defined by Hadash et al. (2017). They specifically describe the inclination to distance oneself from inner experiences, such as thoughts and emotions, which subsequently facilitates reduced reactivity to these internal experiences. In summary, the EQ-D yielded two dimensions whose item content is largely consistent with prior empirical research and theoretical considerations. It can be stated that, in addition to aspects of decentering, the EQ-D also captures aspects of self-compassion and self-acceptance. In line with this, the AS factor was significantly higher associated with self-esteem than the DP factor.

Importantly, it should be noted that Teasdale, Segal and Williams conceptualized the EQ-D with three decentering facets in mind: “the ability to view one’s self as not synonymous with one’s thoughts (e.g., “I can separate myself from my thoughts and feelings”), the ability not to habitually react to one’s negative experiences (e.g., “I can observe unpleasant feelings without being drawn into them”), and the capacity for self-compassion (e.g., “I am better able to accept myself as I am”)” (Fresco et al., 2007, p. 3). Maybe the two-factor solution in our current study aligns more closely with the three decentering facets initially conceptualized by the authors than the 1-factor solution found by Fresco et al. (2007). Based on the item contents (e.g., *I am better able to accept myself as I am*), we presume that self-compassion is primarily reflected in the *Accepting Self-perception* factor. The literature generally agrees that decentering and self-compassion are separate but interrelated constructs, both with positive impacts on well-being (Linardon, 2020; Pérez-Aranda et al., 2021; Biehler and Naragon-Gainey, 2022). In their 2022 study, Biehler & Naragon-Gainey used ecological momentary assessments to explore the interplay of dispositional self-compassion, momentary mindfulness, and momentary affect in predicting momentary well-being. Contrary to their predictions, dispositional self-compassion did not moderate the relationship between momentary mindfulness and well-being. Instead, the study revealed that mindfulness was strongly associated with decentering in daily life. Additionally, high dispositional self-compassion, momentary mindfulness, and lower momentary negative affect were all significant predictors of increased momentary well-being. In another study, mindfulness and self-compassion were found to predict lower anxiety and depression (Pérez-Aranda et al., 2021). A meta-analysis by Linardon (2020) showed that acceptance, mindfulness, and self-compassion principles can be effectively trained using smartphone apps, with some randomized controlled trials already achieving reduced depressive symptoms. This may offer a way to enhance well-being in the general population and among patients. Further research into the interplay between decentering and self-compassion is needed, and we propose using the EQ-D, which also includes aspects of self-compassion (*Accepting Self-perception*), for this purpose.

The current study was the first to validate the EQ-D using EGA. Network models (such as EGA) treat items as causally autonomous as opposed to classic factor analytic methods that assume that all items measure the same latent construct. The goal of network models is to represent a construct (e.g., decentering) as comprehensively as possible with a broad range of items. Christensen et al. (2023) recommend performing a redundancy analysis before EGA to identify highly correlated and potentially redundant items, often caused by shared residual variance (e.g., because the items use very similar wording). Interestingly, item 3 and 14 were identified as redundant item pair in the current study. In the analysis by Fresco et al. (2007), a correlated residual was added to these items. Considering the item content, it is conceivable that these items may also encompass elements of self-compassion, in addition to decentering which could potentially account for the observed high residual correlation. Ignoring redundancies can affect the estimation of dimensions and interpretation of test scores not only in EGA but also in factor models and are thus important to take into account (Christensen et al., 2021). The utilization of the EGA is a strength of the present study, as the EGA is described as a valid and robust method for investigating the dimensionality of questionnaires and has also been previously used in other studies (e.g., Santiago et al., 2021; Laskowski et al., 2023). In addition to its methodological advantages (e.g., redundancy analysis, visual representation of factor structure), EGA is more aligned with current theoretical considerations that view psychological constructs not as latent variables but as a constellation of observed variables (e.g., symptoms; Christensen et al., 2020b). Thus, EGA provides a possible interface between research and practice.

As a limitation of the current study, it should be noted that participants with lower educational attainment (n = 21) were rather underrepresented in the sample which limits the generalizability of the results. Moreover, participants were recruited online which can potentially compromise data integrity. The data integrity checks, however, suggest that the participants completed the questionnaires conscientiously. As a further limitation of the study, it should be noted that no additional questionnaires for assessing decentering, as well as related constructs (aside from the Rosenberg Self-Esteem Scale), and constructs distinct from decentering (aside from the rumination items of the EQ), were included. Therefore, the convergent and discriminant validity of the two-factor structure of the EQ-D as measured in this study should be validated using additional questionnaires. Possible questionnaires for assessing decentering and related constructs such as mindfulness or defusion can for instance be found in Naragon-Gainey and DeMarree (2017) and Hadash et al. (2017). Beyond that and in view of the theoretical considerations and developments in recent years, we suggest to use a multimethod approach to assess decentering in future studies. This is a recommended approach in psychology, as psychological constructs are complex and hardly any questionnaire can capture a construct in its entirety (Eid and Diener, 2006). Recently, Hanley et al. (2020) have for instance developed a decentering questionnaire based on the metacognitive processes model of decentering by Bernstein et al. (2019) which might capture different aspects of decentering than the EQ-D. For future studies, it would be interesting to investigate which clinical outcomes can be predicted specifically by the EQ-D, and which can be better predicted by other questionnaires.

To validly capture constructs, it is essential to ensure that when utilizing questionnaires, these instruments are validated not only based on their theoretical foundation but also through validation with large and diverse samples. The EQ-D has already been validated using various healthy and clinical samples and is the most frequently used questionnaire for the assessment of decentering (Naragon-Gainey et al., 2020). The current study used a large sample with a broad age range to validate the factor structure of the EQ-D using EGA. This approach offered a new perspective on decentering, which, as we hope, will provide a better understanding of the construct for both research and practice. The two dimensions *Distanced Perspective* and *Accepting Self-perception* showed good structural and item consistency and good discriminant validity with rumination. In line with theoretical reasoning and results of previous studies, the *Accepting Self-perceptions* factor showed a significantly higher correlation with self-esteem than the *Distanced Perspective* factor. The results of the current study suggest that the EQ-D comprises aspects of self-compassion next to decentering, both of which are highly relevant constructs for well-being. The EQ is thus a valuable instrument with good psychometric properties, as demonstrated in the present study.

## Data availability statement

The datasets presented in this study can be found in online repositories. The names of the repository/repositories and accession number(s) can be found at: Open Science Framework, https://osf.io/62c3r/.

## Ethics statement

The studies involving humans were approved by ethics committee of the university hospital RWTH Aachen, Germany. The studies were conducted in accordance with the local legislation and institutional requirements. The participants provided their written informed consent to participate in this study.

## Author contributions

LR, BD, SF, SG, and VM conceptualized and designed the study. LR collected and analyzed the data and wrote the original draft. BD, SF, VM, and SG reviewed and edited the manuscript. All authors reviewed the results, contributed to the article, and approved the submitted version.

## Footnotes

^1^ https://qualtrics.com/

^2^ https://osf.io/62c3r/
