# Corrigendum: Validation of the factor structure of the Experiences Questionnaire using Exploratory Graph Analysis

**DOI:** 10.3389/fpsyg.2025.1565123

**Published:** 2025-02-11

**Authors:** Lena Rader, Barbara Drueke, Saskia Doreen Forster, Siegfried Gauggel, Verena Mainz

**Affiliations:** Institute of Medical Psychology and Medical Sociology, University Hospital RWTH Aachen, Aachen, Germany

**Keywords:** decentering, metacognition, experiences questionnaire, exploratory graph analysis, psychometric properties

Due to an oversight, in the original article, there was a mistake in the **Author contributions** statement and LR, BD, SF, and VM were accidentally credited with the preparation of the first draft of the manuscript. The corrected Author contributions statement appears below:

