# Supplementary material for: Validation of the factor structure of the Experiences Questionnaire using Exploratory Graph Analysis
Source: Front Psychol. 2023 Nov 15;14:1250802. doi: 10.3389/fpsyg.2023.1250802 (PMC10684915; doi:10.3389/fpsyg.2023.1250802)
Supplement: Supplementary file 1 [file Data_Sheet_1.pdf]

Supplementary Material

Validation of the Factor Structure of the Experiences Questionnaire using Exploratory Graph Analysis

Lena Rader[1]* , Barbara Drueke[1], Saskia Doreen Forster[1], Siegfried Gauggel[1], & Verena Mainz[1]

***Correspondence**: Lena Rader: lrader@ukaachen.de

1 Supplementary Tables

Table S1:

Descriptive statistics and item content of the Experiences Questionnaire

Item	EQ scale	Mean	SD	Var	Item content
1	Rum	3.85	0.81	0.65	I think about what will happen in the future.
2	Dec	2.95	0.99	0.98	I remind myself that thoughts aren't facts.
3	Dec	3.41	1.02	1.04	I am better able to accept myself as I am.
4	Rum	3.80	0.87	0.76	I notice all sorts of little things and details in the world around me.
5	Dec	2.72	0.99	0.98	I am kinder to myself when things go wrong.
6	Dec	2.83	1.00	1.01	I can slow my thinking at times of stress.
7	Rum	3.08	1.14	1.30	I wonder what kind of person I really am.
8	Dec	2.80	1.00	0.99	I am not so easily carried away by my thoughts and feelings.
9	Dec	2.77	1.00	0.99	I notice that I don't take difficulties so personally.
10	Dec	2.85	1.00	1.00	I can separate myself from my thoughts and feelings.
11	Rum	3.70	0.93	0.87	I analyze why things turn out the way they do.
12	Dec	3.42	0.84	0.70	I can take time to respond to difficulties.
13	Rum	3.53	1.10	1.21	I think over and over again about what others have said to me.
14	Dec	3.26	0.95	0.89	I can treat myself kindly.
15	Dec	3.02	0.92	0.85	I can observe unpleasant feelings without being drawn into them.
16	Dec	3.63	0.87	0.75	I have the sense that I am fully aware of what is going on around me and inside me.
17	Dec	3.11	0.98	0.96	I can actually see that I am not my thoughts.
18	Dec	3.47	0.96	0.92	I am consciously aware of a sense of my body as a whole.
19	Rum	3.36	1.02	1.05	I think about the ways in which I am different from other people.
20	Dec	3.58	0.92	0.85	I view things from a wider perspective.

Note. SD = Standard devation; Var = Variance

2 Supplementary Figures

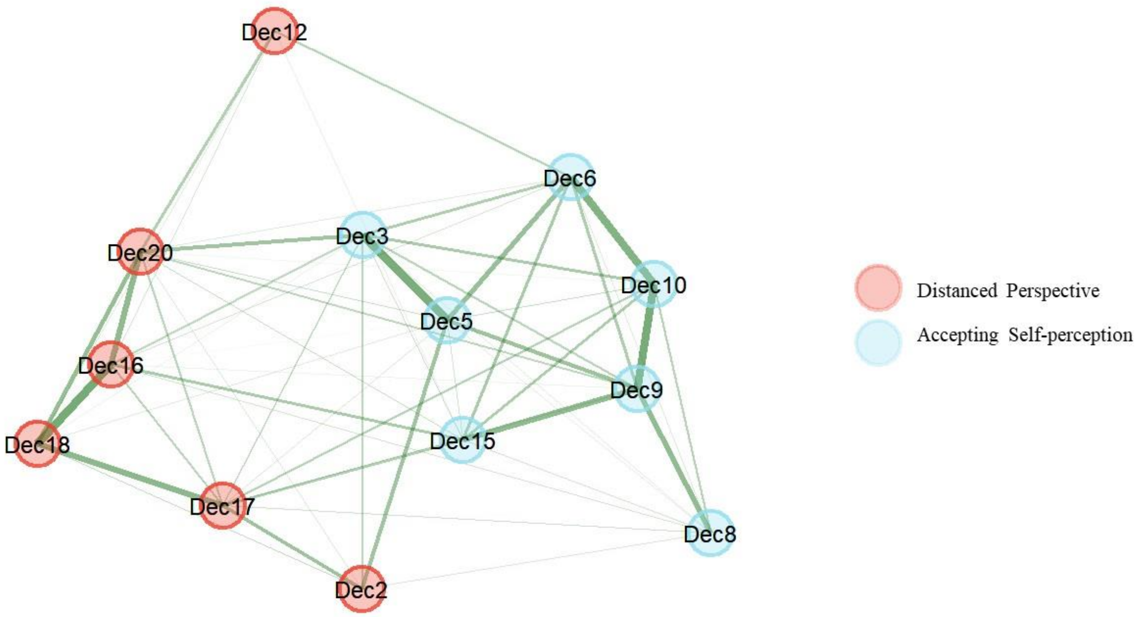

Figure S1. Typical graph of the Decentering subscale of the EQ across all 1000 bootstrap samples of EGA results (excl. item 14)

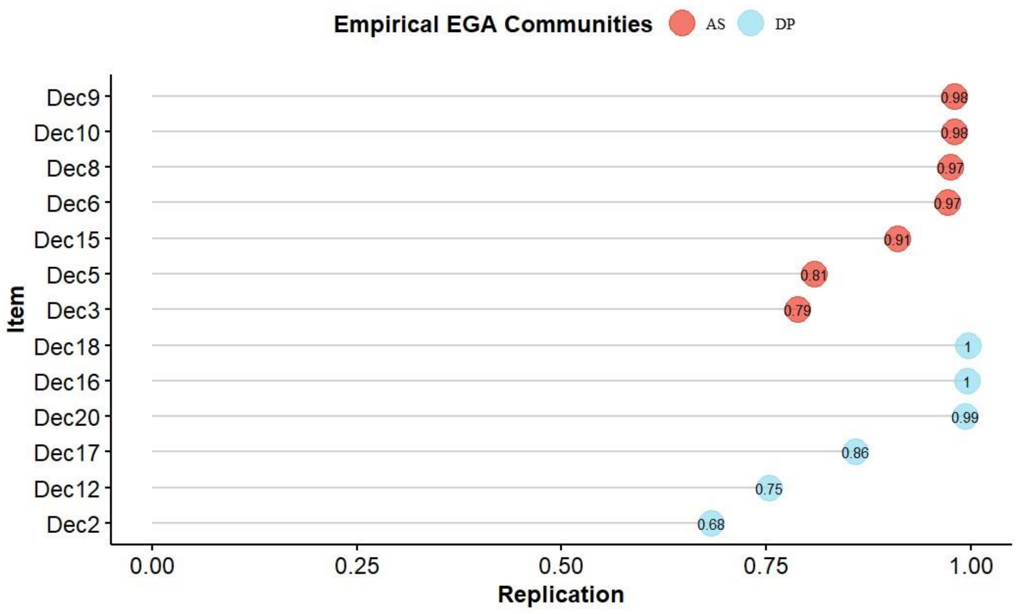

Note. AS = Accepting Self-perception; DP = Distanced Perspective.

Figure S2. Item stability plot of the Decentering subscale of the EQ based on bootstrapped EGA results (excl. item 14)
